# Heterogeneity in mathematics: Investigating cognitive profiles and reading comorbidities among children

**DOI:** 10.1007/s00426-026-02253-1

**Published:** 2026-02-28

**Authors:** Sonia Hasson, Sarit Ashkenazi

**Affiliations:** 1 The Department of Learning Disabilities, The Seymour Fox School of Education, The Hebrew University of Jerusalem, Jerusalem, Israel; 2 The Department of Special Education, Oranim Academic College of Education, Kiryat Tivon, Israel

**Keywords:** Mathematics, Learning disabilities, Heterogeneity in mathematical difficulties, General factors, Specific factors, Mathematical profiles, Comorbidity

## Abstract

Mathematical performance relies on multiple cognitive components, including numerical understanding, language comprehension, and working memory. Although children experiencing mathematical difficulties (MD) share common challenges, their underlying cognitive profiles vary considerably. Understanding this cognitive diversity is important for developing targeted assessment and intervention. This study aims to identify and characterize distinct cognitive profiles among elementary school children, based on their general strengths and weaknesses, and their mathematics-specific cognitive domains. We evaluated 186 children (grades 3–4), including those with MD (*n* = 110), and typically developing peers (*n* = 76) from seven schools in northern Israel. Assessments included domain-specific and domain-general abilities, as well as mathematical and reading performance. Using cluster analysis, we identified five distinct subgroups: Reading Accuracy Difficulties, Mild Mathematical Deficit, High Mathematical Performance, Inhibition Difficulties, and Average Mathematical Performance with reduced non-verbal reasoning. Interestingly, these subgroups were distinguished mainly by their differences in domain-general, rather than domain-specific abilities, thus highlighting the importance of these cognitive factors in Grades 3–4. These findings reflect the diverse nature of MD and its connection to general cognitive factors, which may assist in developing targeted interventions.

**Supplementary information:**

The online version contains supplementary material available at 10.1007/s00426-026-02253-1.

## Introduction

Mathematics learning difficulties affect 3–8% of schoolchildren, despite their average intelligence (Desoete et al., 2004). According to the DSM-5 (American Psychiatric Association, 2013), a specific learning disorder with impairment in mathematics is diagnosed when mathematical skills are substantially below age expectations on standardized tests, persist for at least six months, and interfere significantly with academic or daily functioning.

The underlying origin of mathematical difficulties (MD) remains under discussion in the research field, as some studies argue that domain-specific abilities underlie the primary cause (e.g., Butterworth, 2005; Dehaene, 2011), while others propose that domain-general abilities, such as working memory or inhibition, represent the fundamental source of these challenges (e.g., Passolunghi & Siegel, 2001; Szűcs et al., 2013). However, growing evidence suggests that MD are heterogeneous, with some researchers identifying distinct subtypes that may stem from varying origins of domain-specific numerical deficits, and domain-general cognitive weaknesses (e.g., von Aster & Shalev, 2007; Chan & Wong, 2020).

The current study aims to identify subgroups of children with MD, based on their cognitive profiles. Several theoretical approaches have been proposed to explain the cognitive foundations of mathematical performance.

### The domain-specific theory

The domain-specific approach suggests that mathematical struggles stem from deficits in fundamental numerical skills, particularly in number sense and magnitude representation (Halberda & Feigenson, 2008; Halberda et al., 2008; Libertus et al., 2011; Mazzocco et al., 2011). According to this perspective, math disorders originate from impairments in numerical processing (Butterworth, 2005; Dehaene et al., 2003).

Research has identified several key components that comprise the numerical processing system. The *exact magnitude system* enables rapid and accurate enumeration of small sets without counting (subitizing), a skill observed in infants and non-human species, that strongly predicts later mathematical achievement (Butterworth, 2005; Piazza et al., 2002). The approximate number system (ANS) helps discriminate larger quantities based on Weber’s Law, whereby accuracy depends on the ratio between numbers, rather than their absolute differences (Halberda & Feigenson, 2008). Mathematical ability also depends on symbolic-magnitude mapping—the skill of linking numerical symbols to their actual quantities (Dehaene, 2004)—and on explicit knowledge of the number system, such as placing numbers accurately on a mental number line, which then supports more advanced mathematical reasoning (Geary, 2013; Siegler & Booth, 2004).

Children with MD often exhibit weaknesses across these components, including slower subitizing, reduced ANS acuity, difficulties in symbolic processing, and inaccurate number line estimation (Butterworth, 2005; Chan & Wong, 2020).

### The domain-general theory

While domain-specific theories focus on numerical processing deficits, a substantial body of research documents the influence of domain-general abilities on mathematical performance. Agostini et al. (2022) conducted a comprehensive review examining domain-general cognitive skills in children with MD, based on 46 studies. Their findings revealed consistent impairments in working memory (both verbal and visuospatial), processing speed (particularly in visual search and rapid naming tasks), attention, and executive functions such as inhibition and cognitive flexibility.

Consistent with these findings, Peng et al.‘s (2016) meta-analysis found strong associations between working memory (verbal, numerical, and visuospatial) and mathematical performance, especially among students with learning difficulties, since working memory has been documented as playing a crucial role in mathematical ability (Geary, 1993; Landerl et al., 2009; Peng et al., 2018; Swanson & Siegel, 2001).

Visuospatial working memory supports the solution of mental arithmetic problems by guiding spatial procedural step sequences, and helping to identify relevant information to reach solutions (Caviola et al., 2014; Li & Geary, 2017). It also supports visualization tasks, such as imagining numbers on a number line, understanding relationships between functions, and grasping concepts like place value (Rourke, 1993; Rourke & Finlayson, 1978; Szűcs et al., 2013). At the same time, verbal working memory contributes to mathematical performance by retrieving arithmetic facts, performing mental calculations, and developing conceptual understanding (De Smedt et al., 2010). This system allows for the temporary storage of information while retrieving data from long-term memory, which is important for complex calculations (Geary, 1993). Verbal WM also affects reading, including decoding, reading speed, and comprehension (Swanson & Berninger, 1995).

Furthermore, studies highlight the importance of inhibition – i.e., the ability to delay a response – in mathematical performance (Spiegel et al., 2021; Szűcs et al., 2013). Difficulty in delaying responses may hinder numerical operations, which rely on both temporary and spatial coordination. Szűcs and colleagues (2013) found that impaired visuospatial working memory and impaired delayed response are associated with MD.

Language abilities also influence mathematical performance, and serve as a common underlying factor contributing to the comorbidity of reading and mathematics difficulties (Geary & Hoard, 2001; von Aster & Shalev, 2007). Specifically, language skills affect various mathematical processes such as counting, which provides a foundation for developing arithmetic skills later on (Geary & Hoard, 2001; Göbel & Snowling, 2010; Hanich et al., 2001; Miles et al., 2001; Moll et al., 2015; Simmons & Singleton, 2006). Verbal fluency, particularly the speed at which mathematical facts are retrieved, is important for mathematical performance. Research shows correlations between rapid automatized naming (RAN) and the efficiency of retrieving mathematical facts (Balhinez & Shaul, 2019; Koponen et al., 2007), as well as reading fluency (Koponen et al., 2018).

Fluid intelligence also strongly predicts mathematical abilities (Primi et al., 2010), with longitudinal studies demonstrating its significant capacity to predict future mathematical achievement (Bunge & Vendetti, 2017). This cognitive ability correlates with mathematical tasks that involve reasoning, problem-solving, and relational thinking (Halford et al., 1998; Miller et al., 2014). Research has shown that children with higher fluid intelligence tend to achieve better mathematical outcomes (Salvador et al., 2019), whereas those with lower fluid intelligence encounter greater challenges in mathematics (Archibald et al., 2013; Bartelet et al., 2014).

Although all the cognitive skills discussed in this review are important for mathematical development, the range of MD and their different manifestations suggests that no single factor can be identified as the core deficit behind these challenges. To address this heterogeneity, various models have been developed to explain how domain-general and domain-specific abilities influence mathematical performance. von Aster and Shalev (2007) presented the relationship between domain-specific mathematical skills, which are innate, and domain-general abilities, such as language and working memory, that support the development of higher-level mathematics. Difficulties in any of these abilities can lead to math challenges, with the most severe difficulties occurring when there are deficiencies in the innate, domain-specific abilities.

An alternative theoretical framework suggests that MDs are diverse, and arise from different combinations of cognitive abilities that exist on a continuum, rather than as separate categories (Kroesbergen et al., 2022). Based on this view, both domain-specific and domain-general abilities influence performance in various ways, and cognitive strengths can help compensate for weaknesses.

On the other hand, this complexity has also prompted researchers to identify subtypes, classifying students into different profiles, based on their cognitive traits (See Chan & Wong, 2020; Jordan et al., 2002; Kosc, 1974; Mazzocco & Myers, 2003; von Aster, 2000; von Aster & Shalev, 2007; Salvador et al., 2019), to better understand the heterogeneity of MD; and to improve possible interventions (Mazzocco & Myers, 2003; Salvador et al., 2019; Wilson & Dehaene, 2007).

Next, we examine different methodological approaches for characterizing cognitive profiles in mathematics, and review studies that have identified distinct subgroups among children with diverse mathematical abilities.

### Subtypes of mathematical difficulties

Several approaches have been proposed to classify mathematical difficulties into distinct subgroups. A top-down approach involves proposing theoretical subtypes, and classifying children according to these definitions (Geary, 1993; von Aster, 2000). Based on this research method, Geary (1993) identified three subtypes of MD, each characterized by specific cognitive abilities: (1) The semantic memory subtype, which reflects limitations in long-term memory systems, leading to challenges in retrieving arithmetic facts, and also showing comorbidity with reading difficulties; (2) The procedural subtype, which involves difficulties in executing arithmetic procedures, due to a low capacity for executive working memory; and (3) The visual-spatial representation subtype, resulting in less precise problem-solving strategies. Geary’s classification was based on a comprehensive literature review, examining domain-general cognitive abilities such as working memory, processing speed, and hemispheric brain functions. However, domain-specific mathematical skills were not examined.

Further, von Aster (2000) proposed a theoretical model of developmental dyscalculia, based on empirical findings with children aged 8–11. He identified three subtypes: a verbal subtype, linked to language-related deficits affecting counting and arithmetic fact retrieval; an Arabic numeral subtype, associated with visuospatial-symbolic processing difficulties in reading, writing, and comparing numerals; and a pervasive subtype, the most severe profile, characterized by widespread impairments across mathematical tasks, and attributed to deficits in domain-specific numerical abilities (number sense).

Subsequent research by Bartelet et al. (2014), Salvador et al. (2019), and Chan and Wong (2020) employed a bottom-up approach, using cluster analysis on cognitive and mathematical data to identify naturally occurring groups.

Bartelet et al. (2014) identified six cognitive subtypes among children with MD, in grades 3–6. Three subtypes reflected domain-specific numerical deficits. The “weak mental number line” group showed impaired spatial number representation. The “weak approximate number system” group demonstrated difficulties with non-symbolic magnitude processing. The “access deficit” group was characterized by severe deficits in counting and knowledge of Arabic numerals, exhibiting the most severe arithmetic impairments, particularly in multiplication. This group was the largest in the clinical sample, and showed the most severe mathematical impairments. Two additional subtypes stemmed from domain-general deficits: the “spatial difficulties” group exhibited visuospatial working memory impairments and deficits in approximate numerical knowledge. The “garden-variety” group displayed below-average general intelligence, but did not exhibit a specific numerical-processing weakness. The sixth group performed within the average range on all cognitive measures, despite meeting the criteria for MD, suggesting that non-cognitive factors, such as education or motivation, may underlie their struggles.

The latter comprehensive study provides important evidence for cognitive subtypes. However, its examination of arithmetic focused solely on computational fluency across a relatively broad age range, without distinguishing between pure and comorbid cases, indicating the need for further research.

More recent research by Salvador et al. (2019) investigated cognitive abilities and math performance among children aged 8 to 11, both with and without math difficulties, identifying four subgroups. Two groups with severe MD exhibited poor arithmetic performance on both simple and complex arithmetic tasks, but differed in their cognitive profiles. One group showed general visuospatial deficits, including difficulties with working memory and visuospatial processing, while their number skills remained intact. The other group struggled with numerical magnitude, yet had preserved cognitive abilities. A third group performed at an average level, with mild phonological working memory deficits, and specific challenges in complex calculations. The fourth group excelled across all cognitive and mathematical domains, showing superior intelligence and working memory. Overall, this study showed that similar mathematical challenges may arise from different cognitive pathways, underscoring the complexity of identifying primary sources of deficit in mathematical learning disorders.

An additional study by Chan and Wong (2020) examined subgroups of students with MD, defining five distinct subgroups: three were characterized by specific cognitive deficits (number sense, numerosity coding, and symbolic processing), and another by general cognitive difficulties (both visuospatial and verbal working memory deficits). A fifth subgroup showed mathematical difficulties, without any cognitive impairments.

Current evidence indicates that there are different subgroups within mathematical difficulties, with potential sources of difficulty arising from various cognitive pathways. Despite differences in their methodological approaches, participant characterization, age ranges, and assessment tools, consistent patterns emerge from these studies. These include general cognitive impairments, especially language difficulties (Geary, 1993; von Aster, 2000; Bartelet et al., 2014; Chan & Wong, 2020), working memory deficits—though the specific modalities differ between studies (Geary, 1993; Bartelet et al., 2014; Salvador et al., 2019)—and domain-specific mathematical difficulties, such as deficits in number sense and magnitude processing, which are consistently linked to severe mathematical impairments (von Aster, 2000; Bartelet et al., 2014; Salvador et al., 2019; Chan & Wong, 2020).

The current study aims to identify and characterize distinct cognitive subgroups among students with and without mathematical difficulties, using comprehensive cognitive assessments. Given the documented variability in mathematical learning difficulties, we aim to identify specific cognitive profiles underlying different patterns of mathematical struggles, and to understand how these profiles differ from typical mathematical development.

### Research objectives and hypotheses

Research in the field has documented that MDs are heterogeneous (Bartelet et al., 2014; Salvador et al., 2019; Chan & Wong, 2020). To identify consistency in students’ difficulties, and to develop more tailored assessment and intervention procedures, studies have attempted to categorize these difficulties into subgroups. This approach identifies core deficits within each subgroup, and examines their impact on mathematical abilities (Salvador et al., 2019). However, this area of research continues to develop, and further studies may help refine our understanding of subgroups among struggling students.

In this study, we examined third- and fourth-grade children, with and without MD, to identify their cognitive profiles, with a focus on the cognitive factors underlying their difficulties. These grade levels were selected because mathematical difficulties tend to become more noticeable at this developmental stage, thereby facilitating the identification of specific learning patterns (Geary, 2004). Additionally, research suggests that comorbidity between reading and mathematical difficulties stabilizes around these grades (Koponen et al., 2018).

Building on existing research, we hypothesize that:

H1: Based on research indicating diverse cognitive profiles among MD (Bartelet et al., 2014; Chan & Wong, 2020; Salvador et al., 2019), the study will identify different subgroups of students with varying cognitive profiles.

H2: A subgroup defined by specific mathematical challenges will be identified, displaying deficits in domain-specific skills, such as numerical magnitude comparison, and demonstrating extensive difficulties across various mathematical domains (von Aster, 2000; Bartelet et al., 2014; Salvador et al., 2019).

H3: Subgroups with general cognitive difficulties will emerge, characterized by specific deficits, including language difficulties (which may be associated with reading difficulties) (Geary, 1993; von Aster, 2000), working memory impairments (Bartelet et al., 2014; Chan & Wong, 2020; Salvador et al., 2019), difficulties with inhibition (Szűcs et al., 2013), and lower cognitive ability (Bartelet et al., 2014).

H4: A subgroup of students experiencing mathematical difficulties without cognitive deficits will be identified (Bartelet et al., 2014).

H5: By including students without MD, we expect to identify a subgroup characterized by typical mathematical performance and preserved cognitive skills, as reported by Salvador et al. (2019).

## Methodology

### Participants

Students from seven mainstream elementary schools in northern Israel participated in this study. All participants were Hebrew speakers. Assessment materials, including mathematics and reading tests, were administered in Hebrew, using standardized Hebrew versions of these measures.

After receiving each principal’s approval to participate in this project, teachers at each school were asked to select 3rd and 4th-grade students for the study. The selection of students with MD followed a two-stage process. In the initial stage, teachers identified students who had exhibited persistent difficulties in mathematics, for at least 6 months, consistent with DSM-5 criteria. Identification by teachers relied solely on self-reports, with no standardized questionnaire or structured assessment tool administered. In the second stage, these students’ performance was evaluated using the RAMA test (Authority for Measurement and Evaluation), a national standardized assessment administered throughout Israeli schools. Students who scored at or below the 20th percentile on this assessment were included in the study. This cutoff has been used in previous research to identify MD (e.g., Geary et al., 2000; Passolunghi et al., 1999). Children with documented attention disorders, emotional difficulties, or broader neurodevelopmental diagnoses were excluded from the sample, based on teacher reports and school records.

In the following step, all children completed the Aleph-Taf reading test (Shany et al., 2006), a standardized diagnostic assessment for identifying reading difficulties in Hebrew. Children whose reading accuracy and/or fluency scores were 1 SD or more below the mean, as defined by the test manual criteria, were classified as having reading difficulties.

Teachers were also asked to select typically developing children from the same classrooms, with average or above-average mathematical performance and no reported learning difficulties.

Additionally, all children completed standardized reading assessments. Based on those results, children whose reading accuracy and/or fluency scores were 1 standard deviation or more below the mean, as defined by the test manual criteria, were classified as having reading difficulties.

A total of 189 children participated in this study, including 86 students (51 girls) from third grade (average age: 8.7 years, SD = 0.50) and 100 students (67 girls) from fourth grade (average age: 9.86 years, SD = 0.57). The sample comprised 110 students with MD (50 girls) and 76 typically developing children (68 girls). The final sample comprised 186 participants; two did not complete all tests, and one withdrew for personal reasons.

### Procedure and tools

Our cognitive assessment battery focused on abilities that consistently predict mathematical performance, such as working memory (verbal and visual) (Geary, 2004; Szűcs, 2016), inhibition (Szűcs et al., 2013), language skills (von Aster & Shalev, 2007), and non-verbal reasoning (Primi et al., 2010). We also included foundational numerical skills, identified as prerequisites for mathematical development (Butterworth, 2005). Additionally, we assessed basic mathematical performance, including computational fluency and estimation, as well as reading fluency and accuracy.

All assessments were administered by research team members, including the researcher (doctoral student), research assistants (third-year special education students), or qualified teachers. Prior to data collection, all team members received training with the research instruments and standardized administration procedures, to ensure consistency across all testing sessions.

The assessment sessions were conducted in quiet rooms at the school. The test battery was administered during three sessions, with each session lasting 45 min. All sessions were coordinated fully with the classroom teacher. Children were provided with breaks as needed. Participants who did not complete all assessment sessions were excluded from the final sample.

#### Domain-specific tests

1. **Non-Symbolic Number-Magnitude Comparison**. In this task, two clusters of dots (one in yellow and one in blue) appeared on the screen, each comprising 5–24 dots (ratios of 0.5, 0.55, and 0.45). The participants were then instructed to indicate the larger set of dots by clicking on one of two pre-assigned keys. The stimuli for each trial were presented continuously, until the participant responded. Between trials, a fixation point appeared on the screen for 300ms. The test consisted of six training steps and 48 trials.

The present task shares procedural similarities with dot comparison (Lyons et al., 2014), which demonstrated high internal consistency in prior work (α = 0.977).

(2) **Symbolic Number-Magnitude Comparison**. In this task, two digits between 1 and 9 appeared on the screen, in two different sizes. For example, if the numbers 3 and 8 were displayed on the screen, the *3* may have appeared in a larger format than the *8*. The participants were then instructed to relate only to the actual absolute number, not the physical size of the digits, and choose the larger (higher) number (closest to 10), by selecting one of two keys. Three conditions were manipulated in this study (congruent, natural, and incongruent), with the dependent measure being the average reaction time for each correct response. The stimuli were presented continuously, until the participant responded by clicking one of the two assigned keys. Between the 60 trials, a fixation point appeared on the screen for 500ms. This task was based on the numerical comparison paradigm by Lyons et al. (2014), which has been shown to exhibit high internal consistency in prior work (α = 0.955).

#### Acquired mathematical skills

1. **Arithmetic Fluency. **A pencil-and-paper test was administered to evaluate participants’ mathematical skills. First, they were given 81 written addition problems, each consisting of numbers 1–9, and were asked to answer as many as possible within one minute. Then, they received 81 written multiplication problems involving numbers 1–9, including twin numbers, and were asked to solve as many as possible within a two-minute time limit. Internal consistency between the two subtests was good (α = 0.82). Convergent validity (multiplication–addition) r(198) = 0.72, *p* <.001.
2. **Arithmetic Computation.** Simple addition, multiplication, and subtraction problems were displayed on the computer screen, one at a time, and participants were asked to provide an oral response for each. The reaction time for each correct answer served as the dependent variable. This task was adapted from methodologies used in mathematical cognition research (e.g., Geary et al., 2000) to assess computational fluency and processing efficiency.

Internal consistency across the three arithmetic accuracy indices (addition, subtraction, and multiplication) was good (Cronbach’s α = 0.87).

(3) **Key Math-3 Diagnostic Assessment.** Using the Key Math-3 Diagnostic Assessment (Connolly, 1998), we examined (1) numeration; (2) mental computation and estimation; (3) addition and subtraction; and (4) multiplication and division among the participants. In addition to its high reliability (median subtest-retest reliability = 0.88), this standardized tool was selected for this study because it includes comprehensive mathematical subtests and has already been translated into Hebrew and validated in a previous study (Silverman & Ashkenazi, 2022). The researcher proceeded to the next subtest after the participant had made four consecutive errors, or had answered all problems. Numeration problems (49 questions) and mental computation problems (40 questions) were presented in writing, but answered orally, while addition and subtraction problems (35 items) and multiplication and division problems (31 items) were administered in pencil-and-paper tests. The questions in each subset were progressively more difficult, ranging from simpler to more complex, and each subset yielded a raw score (the absolute number of correct answers) and a standardized score based on grade-level norms.

#### Domain-general tests

1. **Vocabulary. **A total of 23 words were presented orally to participants using the Vocabulary subtest of the Wechsler Intelligence Scale. After hearing each word, they were asked to explain its meaning. This standardized measure assesses verbal intelligence through general vocabulary words of increasing difficulty. The words represent general language development appropriate to the participants’ age. Scores were assigned to each answer, ranging from 0 to 2, based on standardized scoring criteria for definition accuracy. Test reliability: *r* =.76 (Williams et al., 2003).
2. ** Spatial Short-Term and Working Memory.** A computerized Corsi Block Test was used, with nine blue squares (2 cm × 2 cm) unevenly distributed over a 16 cm × 16 cm quadrant (Mueller & Piper, 2014). In each trial, a random sequence of squares was illuminated in yellow, at a rate of one square per second. Following each pair of successful trials, the number of illuminated squares increased gradually from the initial two. The squares remained on the screen for 500 ms, followed by a black screen for 15 s. The same nine blue squares then reappeared on the screen, and the participant had to reproduce the sequence in which the squares had lit up, using the mouse – either in the same order (STM), or in the exact reverse order (WM). Previous evidence supporting the validity and reliability of this task was obtained from Kessels et al. (2000), De Renzi et al. (1977), and Paula et al., (2016).
3. **Verbal Short-Term and Working Memory.** A computerized digit-span test was used to measure the participants’ phonological span (Wechsler, 2003). In each trial, a sequence of numbers was presented orally, and the participant was then asked to repeat the sequence, either in the same order (short-term memory) or in reverse order (working memory). The trials began with a sequence of two numbers, and gradually increased in difficulty with each successive trial. The internal consistency reliability of this task in the current sample was α = 0.73.
4. **Inhibition.** Five fish, lined up in a row, were presented on the screen; the participant was then asked whether the middle fish pointed to the left or the right, regardless of the directions of the other four fish. In the congruent condition, all fish were pointing in the same direction, while in the incongruent condition, the central fish was pointing in one direction, and the remaining four fish were all pointing in the other direction. Each trial began with a 500-ms fixation cross. The fish then remained on the screen until the participant indicated “left” or “right” using the mouse. A 500-ms blank screen followed each trial.

This task was developed based on the established child-friendly flanker paradigm (Rueda et al., 2004).

(5) **Processing Speed.** In this task, participants receive a table in which the numbers from 1 to 9 are assigned to specific abstract symbols. During each trial, a number and a symbol appear on the screen, and participants had to indicate whether the pair was formed, as shown in the table. This task was adapted from the Symbol Digit Modalities Test paradigm (Smith, 1982).
(6) **Non-verbal IQ test.** Using the Raven Color Matrix Test (Raven et al., 1990), participants were asked to complete three consecutive matrices, each consisting of 12 items, yet with increasing difficulty. In each test, the final row of the matrix was missing, and the participant had to select one of six options to complete the matrix correctly. One point was given for each correct answer. This test has demonstrated good reliability, with an internal consistency of 0.89 and split-half reliability of 0.91 (Cotton et al., 2005).
(7) **Verbal fluency test**. Participants were asked to say aloud words for three phonological stimuli (e.g., words beginning with a specific letter) and three semantic stimuli (e.g., names of animals) within 30 s for each stimulus. This task assesses both semantic and phonological fluency. The experimenter documented the students’ responses. The total number of words produced was scored based on age-appropriate norms. The verbal fluency measure was based on the Hebrew fluency tasks by Kavé, following the published procedures and scoring guidelines (Kavé, 2006). Internal consistency between phonological and semantic fluency was (α = 0.70).
(8) **Reading test.** To examine participants’ reading rate and accuracy, they were asked to read words and nonwords using a standardized Hebrew test appropriate to their class level. According to the test manual, the word reading accuracy subtest demonstrates good internal consistency (Cronbach’s α = 0.85), and the reading fluency measure shows high internal consistency (Cronbach’s α = 0.90) (Shany et al., 2006).

### Data analysis

Prior to analysis, all test scores were standardized. For tests with established norms (e.g., KeyMath-3, Vocabulary, Raven’s matrices), grade-based standardized scores were used as provided by the test manuals. For experimental tasks without established norms, raw scores were transformed into standardized z-scores within the current sample. The distributional properties of all study measures were examined using skewness and kurtosis indices, and the results are reported in Supplementary Table S1.

For tests that measured response time or speed and yielded negative values for fast performance (e.g., word-reading speed and inhibition response time), scores were reversed (x -1 ) so that higher scores consistently indicated better performance across all measures.

Pearson correlations were computed between specific mathematical abilities and mathematical performance, as well as between general abilities and mathematical performance (Tables 1 and 2). To reduce the number of factors, we combined highly correlated measures (*r* > .6) into single variables. Specifically, we averaged word- and non-word-reading accuracy scores to create reading accuracy (*r* =.664, *p* <.001), and averaged word- and non-word-reading reaction times to create reading rate (*r* =.627, *p* <.001). Inhibition efficiency was calculated by averaging congruent and incongruent flanker reaction times (*r* =.878, *p* <.001). All other variables were retained as individual standardized scores **(**see correlations in Tables 1 and 2**)**.

**Table 1:** Pearson correlations between specific mathematical abilities and mathematical performance

		1	2	3	4	5	6	7	8	9
1	Fluency test	––								
2	Multiplication & division	.457^**^	––							
3	Estimation	0.661^***^	0.535^**^	––						
4	Numeration	0.624^***^	0.438^**^	0.713^***^	––					
5	Non-symbolic discrete_RT	− 0.247^**^	−.090	− 0.236^*^	− 0.256^**^	––				
6	Non-symbolic discrete_ACC	.03	.024	.013	.100	.068	––			
7	Non-symbolic continuous_ACC	.04	.042	.025	.056	−.096	.020	––		
8	Symbolic_ACC	−.013	.054	.041	.107	.104	.018	−.036	––	
9	Symbolic_RT	− 0.185^**^	−.079	− 0.149^*^	− 0.149^*^	.089	.046	−.115		––

*RT * reaction time, *ACC * accuracy

*p < .05, **p < .01, ***p < .001

**Table 2:** Pearson correlations between general abilities and mathematical performance

		1	2	3	4	5	6	7	8	9	10	11	12	13	14	15	16
1	Phonological Fluency	––															
2	Semantical Fluency	.537^**^	––														
3	Vocabulary	.487^**^	.455^**^	––													
4	Word reading RT	.419^**^	.357^**^	.384^**^	––												
5	Word reading ACC	-.331^**^	-.317^**^	-.429^**^	-.492^**^	––											
6	Non-word reading RT	.345^**^	.232^**^	.260^**^	.627^**^	-.399^**^	––										
7	Non-word reading ACC	-.172^*^	-.132	-.163^*^	-.254^**^	.664^**^	-.261^**^	––									
8	Short-term visual memory	.224^**^	.112	.215^**^	.132	-.151^*^	.096	-.172^*^	––								
9	Visual working memory	.207^**^	.123	.239^**^	.095	-.207^**^	-.002	-.163^*^	.509^**^	––							
10	Raven	303**	.189**	.415**	207**	-.310**	.257*	-.252**	.322**	.375**	––						
11	Flanker congruent RT	-.060	-.127	.188^**^	.-054	.122	-.044	-.116	-.236^**^	.207-^**^	-.277^**^	––					
12	Flanker incongruent RT	.109	.168^*^	-.243^*^	-.132	.145^*^	-.096	.114	-.231^**^	-.187^**^	-.231^**^	-.878**	––				
13	Processing speed ACC	.047	.003	.142^*^	.064	-.058	-.018	-.098	.117	.135	.217**	-.041	-0.064	––			
14	Processing speed RT	-.144^*^	.029	-.040	-.101	.029	-.095	-.027	.101	-.031	-0.043	0.077	046.	077.	––		
15	Short-term verbal memory	.338**	298**	.319*	.284**	-.371**	.300**	-.283**	.278**	-.173*	.279**	-.131	-.133	103.	004.	––	
16	Verbal working memory	185**	0.107	200**	.216**	-.224**	.136*	-.153*	.179**	130.	.295**	-.001	0.04	0.14	0.74	^*^172.	––

*RT * reaction time, *ACC * accuracy

*p < .05, **p < .01, ***p < .001

To identify distinct cognitive-mathematical profiles within the heterogeneous sample, we conducted a cluster analysis of 186 participants, using standardized z-scores across a broad set of cognitive and mathematical measures. This approach aimed to identify subgroups of students with similar cognitive and academic profiles, thereby providing a more nuanced understanding of the different manifestations of MD. Cluster analysis assumes that the data contain a meaningful structure that can be organized into relatively homogeneous groups (Bartelet et al., 2014; Chan & Wong, 2020; Salvador et al., 2019).

A two-stage clustering procedure was employed, following established methodology (Bartelet et al., 2014). In the first stage, hierarchical cluster analysis (Ward’s method) was conducted to determine the optimal number of clusters, which indicated six clusters. In the second stage, K-means clustering was applied to assign participants to clusters. One cluster comprised only two participants and was excluded, yielding five final clusters for subsequent analyses.

Following the cluster analysis, we characterized each group’s patterns of strengths and difficulties across cognitive, mathematical, and reading abilities. Performance was classified using standardized criteria: 25th percentile or below (z ≤ −0.67) indicated difficulty, 20th percentile or below (z ≤ −0.84) indicated significant difficulty, and 16th percentile or below (z ≤ −1.0) indicated severe difficulty.

To examine whether the clusters differed significantly on key variables, we conducted one-way ANOVAs across all measures, followed by Bonferroni-corrected pairwise t-tests (detailed pairwise comparison results are provided in the Supplementary Materials). Additionally, we conducted an ANCOVA, controlling for age to assess whether group differences remained significant.

## Results

The analysis identified six groups. Since one group had only two participants, we proceeded with five groups. We then applied K-means clustering to refine the groupings (see Table 3).

**Table 3:** Mean Z-scores for general and specific cognitive domains across clusters ׁ(S.D.)

	Cluster 1	Cluster 2	Cluster 3	Cluster 4	Cluster 5
	Reading Accuracy Difficulties	Mild Mathematical Deficit	High Mathematical Performance	Inhibition Difficulties	Average Mathematical Performance
	*N* = 41	*N* = 48	*N* = 53	*N* = 7	*N* = 35
Domain-Specific					
Math fluency test	−0.69 (0.58)	−0.34 (0.63)	0.63 (0.84)	−0.64 (0.77)	0.07 (0.81)
Simple operations	−0.89 (0.98)	0.05 (0.83)	0.63 (0.66)	−0.86 (0.96)	−0.05 (0.86)
Addition & Subtraction	−0.71 (0.84)	−0.22 (0.93)	0.51 (0.78)	−0.92 (0.75)	0.22 (0.84)
Multiplication & division	−0.71 (0.71)	−0.32 (0.87)	0.55 (0.83)	−0.30 (0.82)	0.17 (0.85)
Estimation	−0.69 (0.79)	−0.29 (0.88)	0.66 (0.85)	−0.91 (0.70)	0.13 (0.84)
Numeration	−0.64 (0.86)	−0.21 (0.90)	0.61 (0.78)	−0.80 (0.93)	0.07 (0.97)
Non-symbolic comparison –RT	−0.43 (1.06)	0.13 (0.89)	0.29 (0.98)	−0.76 (0.88)	−0.01 (0.86)
Symbolic comparison RT	−0.27 (0.76)	−0.16 (0.93)	0.30 (1.20)	−0.30 (0.94)	0.02 (0.79)
Non-symbolic comparison –ACC	−0.40 (1.08)	−0.14 (1.10)	0.21 (0.94)	0.06 (0.89)	0.17 (0.81)
Number line	−0.52 (1.07)	0.24 (0.76)	0.27 (0.54)	−0.36 (0.86)	−0.03 (0.76)
Domain-General					
Verbal fluency	−0.51 (0.76)	−0.11 (0.88)	0.43 (0.96)	−0.67 (0.58)	−0.14 (0.62)
Reading RT	−0.07 (0.85)	−0.05 (1.08)	0.14 (1.23)	−0.50 (0.59)	−0.13 (0.71)
Reading ACC	−0.59 (1.07)	−0.02 (0.82)	0.38 (0.75)	−0.12 (1.07)	−0.03 (0.81)
Vocabulary	−0.54 (0.70)	−0.16 (0.97)	0.44 (1.01)	−0.93 (0.58)	0.02 (0.91)
Verbal working memory	−0.41 (0.9)	0.01 (0.84)	0.54 (1)	−0.6 (1)	−0.35 (0.88)
Short-term visual memory	−0.35 (0.81)	0.17 (0.82)	0.37 (0.98)	−0.84 (0.77)	−0.20 (1.02)
Visual working memory	−0.52 (0.78)	0.18 (0.85)	0.33 (1.04)	−0.45 (0.52)	−0.07 (1.08)
Inhibition	−0.10 (0.77)	0.12 (0.54)	0.03 (0.89)	−5.37 (1.20)	−0.20 (1.10)
Coding	−0.04 (0.27)	−0.13 (0.18)	0.06 (1.15)	0.11 (0.31)	−0.15 (0.22)
Non-verbal IQ	−0.61 (0.78)	0.10 (0.75)	0.67 (0.68)	−0.66 (0.30)	−0.72 (0.79)

*RT * reaction time, *ACC * accuracy

The ANOVA showed significant differences between groups for most mathematical and cognitive measures. The largest effect sizes were observed in inhibition (η² = 0.599), non-verbal IQ (η² = 0.379), math fluency test (η² = 0.337), and simple operations fluency (η² = 0.322), with significant differences also emerging in estimation (η² = 0.292), multiplication & division (η² = 0.26), addition & subtraction (η² = 0.258), numeration (η² = 0.24), and vocabulary (η² = 0.161). By contrast, coding and reading RT did not differ significantly across clusters, while symbolic comparison RT and non-symbolic comparison accuracy showed significant, but small effects (see Table 4).

**Table 4:** One-way ANOVA: Between-cluster comparisons

Variable	Df	F-value	Eta Squared (η²)	*p*-value
Inhibition	F(4, 183)	68.27	0.599	< 0.001
Non-verbal IQ	F(4, 179)	27.28	0.379	< 0.001
Math fluency test	F(4, 182)	23.1	0.337	< 0.001
Simple operations	F(4, 183)	21.73	0.322	< 0.001
Estimation	F(4, 182)	18.72	0.292	< 0.001
Multiplication & division	F(4, 182)	15.96	0.26	< 0.001
Addition & Subtraction	F(4, 171)	14.87	0.258	< 0.001
Numeration	F(4, 183)	14.46	0.24	< 0.001
Non-numerical fluency	F(4, 184)	9.3	0.168	< 0.001
Vocabulary	F(4, 184)	8.85	0.161	< 0.001
Verbal working memory	F(4, 183)	8.8	0.161	< 0.001
Number line	F(4, 184)	7.61	0.142	< 0.001
Reading ACC	F(4, 184)	7.32	0.137	< 0.001
Short-term visual memory	F(4, 184)	6.24	0.119	< 0.001
Visual working memory	F(4, 184)	5.8	0.112	< 0.001
Non-symbolic comparison –RT	F(4, 184)	4.77	0.094	0.001
Non-symbolic comparison –ACC	F(4, 184)	2.79	0.057	0.028
Symbolic comparison –RT	F(4, 181)	2.57	0.054	0.040
Coding	F(4, 178)	0.88	0.019	0.475
Reading RT	F(4, 184)	0.87	0.019	0.484

*ACC * Accuracy, *RT * Reaction Time

The ANCOVA results were consistent with ANOVA findings, with all variables that showed significant differences in the ANOVA remaining significant after age adjustment (Table 5).

**Table 5.** ANCOVA Results (Controlling for Age)

Variable	Df	F-value	Eta Squared (η²)	*p*-value
Inhibition	F(4, 149)	42.03	0.501	< 0.001
Non-verbal IQ	F(4, 146)	19.57	0.292	< 0.001
Math fluency test	F(4, 148)	18.41	0.3	< 0.001
Simple operations fluency	F(4, 149)	17.66	0.279	< 0.001
Estimation	F(4, 148)	18.6	0.279	< 0.001
Multiplication & division	F(4, 148)	14.21	0.239	< 0.001
Addition & Subtraction	F(4, 138)	15.13	0.27	< 0.001
Numeration	F(4, 149)	14.45	0.236	< 0.001
Non-Numerical Fluency	F(4, 184)	9.30	0.168	< 0.001
Vocabulary	F(4, 150)	6.76	0.131	< 0.001
Verbal working memory	F(4, 149)	5.3	0.104	< 0.001
Number line	F(4, 150)	3.87	0.066	0.005
Reading ACC	F(4, 150)	4.29	0.091	0.003
Short-term visual memory	F(4, 150)	3.32	0.071	0.012
Visual working memory	F(4, 150)	5.04	0.094	< 0.001
Non-symbolic comparison –RT	F(4, 150)	3.45	0.067	0.010
Non-symbolic comparison –ACC	F(4, 150)	2.08	0.043	0.086
Symbolic comparison –RT	F(4, 147)	2.33	0.052	0.058
Coding	F(4, 144)	0.99	0.024	0.416
Reading RT	F(4, 150)	2.05	0.042	0.090

*p <.05, **p <.01, ***p <.001

**Fig. 1:**
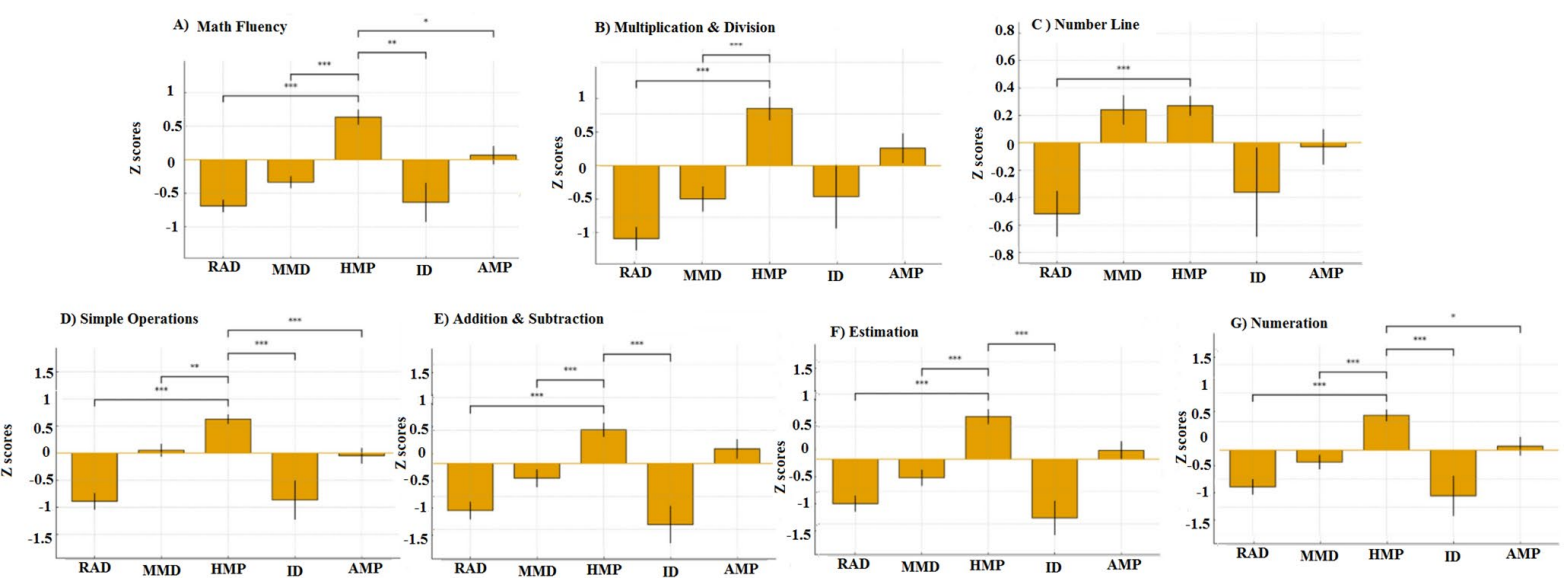
Between Cluster comparisons of Math abilities. RAD = Reading Accuracy Difficulties. MMD = Mild Mathematical Performance. HMP = High Mathematical Performance. ID = Inhibition Difficulties. AMP = Average Mathematical Performance. A) Math Fluency. B) Multiplication & Division. C) Number Line. D) Simple Operations. E) Addition & Subtraction. F) Estimation. G) Nueration. Error bars represent standard error

**Fig. 2:**
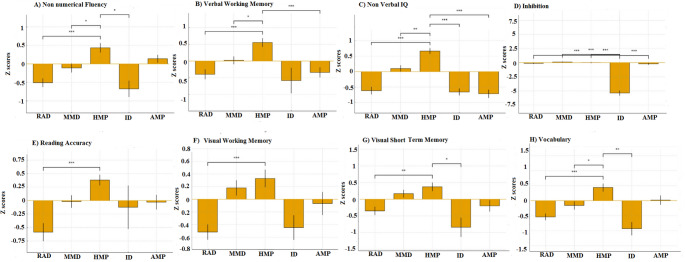
Between-cluster comparisons of domain general abilities. RAD = Reading Accuracy Difficulties. MMD = Mild Mathematical Performance. HMP = High Mathematical Performance. ID = Inhibition Difficulties. AMP = Average Mathematical Performance. A) Non numerical Fluency. B) Verbal Working memory. C) Non verbal IQ. D) Inhibition. E) Reading Accuracy. F) Visual Working Memory. E) Visual Short Term Memory. H) Vocabulary. Error bars represent standard error

The five clusters, ranging in size from 7 to 53 participants, are detailed in the following section. Each cluster demonstrated a distinct cognitive-mathematical profile, characterized by specific patterns of strengths and weaknesses (see Table 3 for mean scores, and Figs. 1 and 2 for a visual comparison between the groups).

### Cluster characterization

#### Cluster 1: Reading accuracy difficulties (*n* = 41)

This group exhibited pronounced difficulties across all mathematical domains, compared to the other clusters. Within this group, mathematical abilities were impaired across nearly all domains, with the most severe deficits in simple operations (*M* = −0.89, *SD* = 0.98), and addition and subtraction (*M* = −0.71, *SD* = 0.84). Several general cognitive abilities were also reduced, with low performance in non-verbal reasoning (*M* = −0.61, *SD* = 0.78), vocabulary (*M* = −0.54, *SD* = 0.70), visual working memory (*M* = −0.52, *SD* = 0.78), and non-numerical fluency (*M* = −0.51, *SD* = 0.76).

Compared to Cluster 3 (no difficulties group), this group performed significantly lower across multiple domains, with especially large differences in simple operations (*d* = −1.70, *p* <.001), estimation (*d* = −1.15, *p* <.001), arithmetic fluency (*d* = −1.20, *p* <.001), vocabulary (*d* = −1.26, *p* <.001), visual working memory (*d* = −0.95, *p* <.001) and reading accuracy (d = −0.89, *p* <.001). Compared with Cluster 2 (light mathematical gaps), they also showed weaker performance on simple operations (d = −0.67, *p* <.001) and nonverbal reasoning (*d* = −0.59, *p* <.01).

This cluster consisted primarily of children with MD (95%). Moreover, 53% of MD children in this group had reading difficulties.

#### Cluster 2: Mild mathematical deficit (*n* = 48)

This group did not exhibit severe mathematical deficits, but their performance was consistently lower, compared to Cluster 3 (typically developing). General cognitive abilities were largely preserved, with scores on vocabulary (M = −0.16, SD = 0.97), verbal working memory (M = 0.01, SD = 0.84), and nonverbal reasoning (*M* = 0.10, *SD* = 0.75) falling within the average range.

Mathematical domains showed modest gaps, specifically in estimation (M = −0.29, SD = 0.88) and multiplication and division (*M* = −0.32, *SD* = 0.87). However, compared with Cluster 3, significant differences emerged, primarily in mathematical measures: simple operations (d = −1.04, *p* <.01), numeration (d = −0.61, *p* <.001), and estimation (d = −0.55, *p* <.01), with no weaknesses in general cognitive abilities.

In this group, 48% of the participants experienced difficulties with math; additionally, 45% of the MD participants showed reading difficulties.

#### Cluster 3: High mathematical performance (*n* = 53)

This cluster included no students with MD. All children demonstrated average to above-average performance across cognitive abilities. Specifically, general cognitive abilities were preserved, with average to high scores, for instance, in non-verbal reasoning (M = 0.67, SD = 0.6) and verbal working memory (M = 0.54, SD = 1). Mathematical performance was comparably strong, showing high performance in estimation (M = 0.66, SD = 0.8), numeration (M = 0.61, SD = 0.7), and arithmetic fluency (M = 0.63, SD = 0.6). Compared to groups with MD, this group exhibited significant advantages across multiple domains.

#### Cluster 4 – Inhibitory control deficit (*n* = 7)

This group was characterized by severely impaired inhibitory control (*M* = −5.37, *SD* = 1.20), standing out as the most impaired domain, relative to all other groups. Students in this cluster showed difficulties across most mathematical skills, including estimation (*M* = −0.91, *SD* = 0.70), math fluency (*M* = −0.64, *SD* = 0.77), numeration (*M* = −0.80, *SD* = 0.93), and addition & subtraction (*M* = −0.92, *SD* = 0.75). Additional weaknesses were observed in verbal domains: vocabulary (*M* = −0.93, *SD* = 0.58), non-numerical fluency (*M* = −0.67, *SD* = 0.58), and reading rate (*M* = −0.50, *SD* = 0.59). Processing speed was also slower on non-symbolic comparison tasks (*M* = 0.76, *SD* = 0.88).

Compared to Cluster 3, children in Cluster 4 performed significantly lower across multiple domains, with significant effects in inhibition (d = −4.75, *p* <.01), vocabulary (d = −1.83, *p* <.001), estimation (d = −1.69, *p* <.001), math fluency (d = −1.44, *p* <.001), addition & subtraction (d = −1.59, *p* <.001, and numeration (d = −1.59, *p* <.001). Most children in this group (85%) met the criteria for MD, and over half (57%) were also identified as having reading challenges.

#### Cluster 5 – Average mathematical performance (*n* = 35)

This cluster (*n* = 35) showed average mathematical performance, with no significant differences from Cluster 3 on mathematical measures. Compared with Cluster 3, there were significant differences in nonverbal reasoning (d = −0.75, *p* <.05), whereas mathematical competencies remained relatively intact, as did other cognitive abilities.

Mathematical difficulties were identified in 40% of children in this cluster, and 11% of them also had reading difficulties.

## Discussion

The cluster analysis identified five distinct subgroups of children, varying in size, prevalence of MD diagnoses, and cognitive and academic profiles. Two of the clusters were associated with severe mathematical learning difficulties, and showed a high percentage of children with a diagnosis of MD. The first cluster (*n* = 41) was characterized by severe learning difficulties, with 95% of participants diagnosed with MD, and 53% also showing reading weakness. Children in this cluster demonstrated broad deficits across domain-general abilities (e.g., non-verbal IQ, verbal and visual working memory, verbal fluency, and reading accuracy) and mathematical skills (e.g., math fluency, operations, estimation, numeration, and number line). This cluster was uniquely associated with inaccurate reading.

The fourth cluster, similar to Cluster 1, showed severe learning difficulties. Although small in size (*n* = 7), it included a high percentage of children with learning difficulties, with 85% diagnosed with MD and 57% also showing reading weakness. This cluster demonstrated a unique and very severe weakness in inhibition. Interestingly, participants in this cluster also showed deficits across most other cognitive and mathematical measures. More pronounced difficulties emerged in tasks associated with basic numerical processing (e.g., numeration and estimation), while difficulties in complex calculations (e.g., multiplication and division) were less severe.

Two of the clusters showed moderate weaknesses. The second cluster (*n* = 48) displayed a mild pattern of mathematical weakness, particularly in fluency, addition and subtraction, and multiplication and division, though less severe than in Clusters 1 and 4. These children performed slightly below average in domain-general abilities, including non-verbal IQ, non-numerical fluency, and vocabulary. Nearly half of the participants in this cluster were diagnosed with MD, and 45% also showed reading deficits.

The fifth cluster (*n* = 35) exhibited mathematical performance mostly within the average range, while showing weaknesses in non-verbal reasoning. Notably, 40% of the children in this cluster were defined as having MD, whereas only 22% also showed reading weakness.

Finally, the third cluster (*n* = 53) demonstrated mathematical performance primarily in the high range, along with strong domain-general abilities. None of the children in this cluster were diagnosed with MD.

Taken together, these results show that mathematical abilities vary from multi-domain impairments (Clusters 1 and 4), to mixed difficulties (Clusters 2 and 5), to no difficulties or even higher abilities (Cluster 3). This pattern of varying levels of MD may also be seen in Geary et al.‘s (2012) research, which identified students with severe and comprehensive mathematical deficits, along with working memory and cognitive impairments (Mathematical Learning Disability - MLD), students with more specific MD, but average cognitive abilities (Low Achieving - LA groups), and typically achieving children.

An additional important finding concerns the relation between math and reading. In the clusters with severe multi-domain impairments (Clusters 1 and 4), comorbidity rates were high (above 50%), demonstrating that reading difficulties frequently co-occur with MD.

This finding is consistent with existing research, indicating that the overlap between reading and math difficulties stems from shared, underlying cognitive abilities affecting both domains (Landerl & Moll, 2010; Moll et al., 2015; Willcutt et al., 2013). Studies have shown that inhibition influences both reading and math challenges (Johann et al., 2019), while verbal working memory is also strongly associated with reading difficulties (Landerl et al., 2009; Swanson & Berninger, 1995).

Verbal fluency, also observed in groups 1 and 4, was also found to be one of the cognitive abilities that affects comorbidity (Koponen et al., 2018). Weaknesses in verbal fluency hinder the development of basic mathematical skills, such as counting procedures, which are crucial for retrieving arithmetic facts (Donlan, 1998; Rousselle et al., 2004; Geary, 2004). Since mathematical facts depend on counting processes, difficulties with verbal fluency may hinder the efficient storage and retrieval of arithmetic facts from long-term memory (Geary, 2004; Siegler & Shrager, 1984). Reading difficulties are also closely linked to fluency deficits, particularly in rapid automatized naming tasks that require rapid access to phonological representations, which may explain the frequent co-occurrence of reading and math difficulties (Koponen et al., 2018).

Another key finding relates to dissociations between domain-general and domain-specific factors. In Cluster 5, a dissociation was found between low, non-verbal IQ, and average mathematical achievement, indicating that domain-general weaknesses do not always fully manifest in mathematical performance.

Previous studies examining subgroups in mathematics have also documented dissociations between non-verbal reasoning abilities and arithmetic performance, particularly in basic computational tasks. More specifically, Bartelet et al. (2014) found that a subgroup characterized by below-average general intelligence, did not exhibit severe arithmetic difficulties, although their study focused primarily on basic computational fluency. Research by Peng et al. (2018) examined the differential effects of fluid intelligence on mathematical performance, finding that these abilities primarily influenced higher-level mathematical tasks such as word problems, while basic computational performance showed weaker associations with general cognitive abilities. On the other hand, Salvador et al.‘s (2019) study showed that high intelligence was associated with superior mathematical performance, in typically developing children. This pattern was also documented in our study, where Profile 3 demonstrated both higher intelligence scores (Raven’s and vocabulary), compared to other groups, and correspondingly superior mathematical performance.

Based on these findings, our research hypotheses receive partial support. The first hypothesis was confirmed, demonstrating that MDs are indeed heterogeneous and influenced by domain-general cognitive abilities. Consistent with the third hypothesis, two distinct subgroups with general cognitive difficulties were identified. The fourth hypothesis was also supported, as one subgroup exhibited mild difficulties, despite intact general cognitive abilities, suggesting that these difficulties may stem from environmental factors such as inadequate instruction, limited educational opportunities, or motivational factors, rather than underlying cognitive deficits (Bartelet et al., 2014), or from deficits in domain-general cognitive abilities, that were not included in our assessment battery.

However, the second hypothesis was not supported. No subgroup defined primarily by deficits in core numerical abilities was identified among students in grades 3–4. This finding suggests that mathematical performance in these elementary grades may depend more heavily on domain-general cognitive abilities, than on fundamental numerical processing skills.

Several previous studies also reported a lack of association between basic numerical and non-numerical processing abilities and arithmetic performance in school-age children (Szűcs et al., 2013; Wilkey & Ansari, 2020). This suggests that domain-general cognitive deficits might underlie mathematical learning difficulties, rather than impairments in core numerical skills. For example, Szűcs et al. (2013) demonstrated that domain-general cognitive factors, such as visuospatial working memory and inhibition, influence low mathematical achievement; however, no relationships were observed between symbolic and non-symbolic representation abilities and mathematical performance. Additionally, Wilkey and Ansari (2020) pointed out in their review that there is no specific neural mechanism dedicated to number sense. Instead, different brain regions that control attention, inhibition, and visual processing influence numerical perception.

An additional explanation for this lack of association could stem from focusing on single-digit numerals, which may be too simple for children at this age. Indeed, Holloway and Ansari (2009) found that the older the child, the weaker the relationship between comparing one-digit numbers and math abilities. Additionally, Nelwan et al. (2022) reported that comparing symbolic representations only predicted difficulties in mathematics among second graders, not among children in higher grades. Future research might benefit from testing larger number comparisons.

## Conclusions

Our study identified five distinct subgroups, characterized by different cognitive difficulties and varied patterns of mathematical performance. Consistent with previous research, our findings confirmed the influence of domain-general cognitive abilities, especially working memory, inhibition, and verbal fluency, on mathematical performance and reading comorbidities. These results concur with earlier studies that have recognized similar cognitive profiles. Bartelet et al. (2014); Salvador et al. (2019), and Chan and Wong (2020) identified subgroups marked by working memory deficits. Research has also shown that verbal fluency affects mathematical abilities (Koponen et al., 2007), supporting our findings, as well as inhibition (Szűcs et al., 2013). Although inhibition has been linked to both mathematical and reading performance, these connections have not been explicitly examined within subgroups of learning difficulty.

These findings highlight the importance of a comprehensive cognitive assessment when supporting children with MD. Rather than focusing solely on mathematical skills, evaluating underlying cognitive strengths and weaknesses across domains such as working memory, inhibition, verbal fluency, and reading may enable more targeted interventions.

### Educational implications

We believe that comprehensive assessment and targeted intervention can support children’s development. Mathematics involves multiple cognitive abilities, so identifying students’ primary challenges, and understanding which abilities most significantly affect their math performance, can help us develop more effective support strategies.

This comprehensive assessment approach would enable educators to distinguish between children who require intensive, multi-domain interventions, and those who would benefit from more targeted skill-building in specific areas.

### Limitations

Several limitations should be acknowledged. First, although our sample of 186 students provided adequate power for cluster analysis, some emerging clusters contained relatively small numbers of participants (the fourth cluster included only seven students), which limits the generalizability and statistical power for drawing robust conclusions about these subgroups. Future research would benefit from larger samples, to ensure adequate representation within each identified cluster.

Second, because the study was conducted after the COVID-19 pandemic, some findings may have been influenced by the shift to remote learning that participants experienced in previous years. Therefore, applying these findings to routine educational contexts should be made with caution.

Third, in order to validate and interpret the clusters, we defined standardized criteria for levels of difficulty: performance at or below the 25th percentile indicated difficulty, at or below the 20th percentile indicated significant difficulty, and at or below the 16th percentile indicated severe difficulty. It should be noted that these ranges are very close to one another, and may not allow for clear differentiation between participants.

Fourth, it has been suggested that the diagnosis of MD should rely on a clinical diagnosis requiring a comprehensive individual assessment. However, in line with prior work (Bartelet et al., 2014; Chan & Wong, 2020), we used a two-stage identification process that included teacher reports and a cutoff on a standardized individually-administered assessment.

Additionally, although we followed established methodology for cluster determination, we did not systematically evaluate alternative clustering solutions (but see Supplementary Material 1 for LCA analysis), or use multiple validation methods. Future research could include formal model comparison criteria, consensus clustering methods, and alternative clustering algorithms.

Furthermore, reliability indices for the non-symbolic and symbolic comparison tasks were not computed for the current sample. These tasks were adapted from well-established paradigms (e.g., Lyons et al., 2014) with demonstrated high internal consistency in prior research; however, future studies should assess reliability within their specific samples.

Lastly, there was a notable gender imbalance in the typical group, which was predominantly female (68 girls, eight boys). Future studies could benefit from greater gender balance across groups.

## Supplementary information

Below is the link to the electronic supplementary material.

Supplementary Material 1 (DOCX 62.8 KB)

Supplementary Material 2 (DOCX 32.4 KB)

## Data Availability

Detailed datasets will be provided upon request from the corresponding author.

## Declarations

### AI assistance statement

In this manuscript, AI assistance was used for language editing and formulation purposes.
