# Supplementary material for: Heterogeneity in mathematics: Investigating cognitive profiles and reading comorbidities among children
Source: Psychol Res. 2026 Feb 28;90(2):47. doi: 10.1007/s00426-026-02253-1 (PMC12950034; doi:10.1007/s00426-026-02253-1)
Supplement: Supplementary file 1 — Supplementary Material 1 (DOCX 62.8 KB) [file 426_2026_2253_MOESM1_ESM.docx]

Supplementary Materials for: Heterogeneity in Mathematics: Investigating Cognitive Profiles and Reading Comorbidities among

Children.

Sonia Hasson, Sarit Ashkenazi

**Table S1** *Pairwise Post-hoc Comparisons (Bonferroni-Adjusted) — Numeric Group Labels*

| Measure | Group 1 | Group 2 | Mean difference (ΔM) | t (df) | p (Bonferroni) |
| --- | --- | --- | --- | --- | --- |
| Inhibition | 1 | 2 | 0.22 | 1.61 (89) | 1.000 |
| Inhibition | 1 | 3 | 0.13 | 0.75 (95) | 1.000 |
| Inhibition | 1 | 4 | -5.26 | -15.29 (46) | < .001 |
| Inhibition | 1 | 5 | -0.09 | -0.43 (73) | 1.000 |
| Inhibition | 2 | 3 | -0.09 | -0.63 (104) | 1.000 |
| Inhibition | 2 | 4 | -5.48 | -20.93 (55) | < .001 |
| Inhibition | 2 | 5 | -0.32 | -1.74 (82) | .857 |
| Inhibition | 3 | 4 | -5.39 | -14.52 (61) | < .001 |
| Inhibition | 3 | 5 | -0.22 | -1.05 (88) | 1.000 |
| Inhibition | 4 | 5 | 5.17 | 11.16 (39) | < .001 |
| Raven | 1 | 2 | -0.71 | -4.38 (87) | < .001 |
| Raven | 1 | 3 | -1.28 | -8.47 (92) | < .001 |
| Raven | 1 | 4 | 0.04 | 0.15 (46) | 1.000 |
| Raven | 1 | 5 | 0.10 | 0.56 (74) | 1.000 |
| Raven | 2 | 3 | -0.57 | -3.97 (99) | .001 |
| Raven | 2 | 4 | 0.76 | 2.62 (53) | .115 |
| Raven | 2 | 5 | 0.82 | 4.76 (81) | < .001 |
| Raven | 3 | 4 | 1.33 | 5.06 (58) | < .001 |
| Raven | 3 | 5 | 1.38 | 8.72 (86) | < .001 |
| Raven | 4 | 5 | 0.06 | 0.18 (40) | 1.000 |
| Non-Numerical Fluency | 1 | 2 | -0.40 | -2.29 (89) | .242 |
| Non-Numerical Fluency | 1 | 3 | -0.94 | -5.22 (95) | < .001 |
| Non-Numerical Fluency | 1 | 4 | 0.16 | 0.54 (46) | 1.000 |
| Non-Numerical Fluency | 1 | 5 | -0.65 | -4.02 (74) | .001 |
| Non-Numerical Fluency | 2 | 3 | -0.54 | -3.03 (104) | .031 |
| Non-Numerical Fluency | 2 | 4 | 0.57 | 1.64 (55) | 1.000 |
| Non-Numerical Fluency | 2 | 5 | -0.25 | -1.43 (83) | 1.000 |
| Non-Numerical Fluency | 3 | 4 | 1.11 | 2.98 (61) | .041 |
| Non-Numerical Fluency | 3 | 5 | 0.30 | 1.63 (89) | 1.000 |
| Non-Numerical Fluency | 4 | 5 | -0.81 | -3.19 (40) | .028 |
| Vocabulary | 1 | 2 | -0.38 | -2.11 (89) | .378 |
| Vocabulary | 1 | 3 | -0.98 | -5.32 (95) | < .001 |
| Vocabulary | 1 | 4 | 0.39 | 1.39 (46) | 1.000 |
| Vocabulary | 1 | 5 | -0.56 | -3.00 (74) | .036 |
| Vocabulary | 2 | 3 | -0.60 | -3.09 (104) | .026 |
| Vocabulary | 2 | 4 | 0.77 | 2.05 (55) | .451 |
| Vocabulary | 2 | 5 | -0.18 | -0.85 (83) | 1.000 |
| Vocabulary | 3 | 4 | 1.37 | 3.49 (61) | .009 |
| Vocabulary | 3 | 5 | 0.42 | 2.00 (89) | .489 |
| Vocabulary | 4 | 5 | -0.95 | -2.63 (40) | .121 |
| Verbal WM | 1 | 2 | -0.05 | -2.21 (89) | .294 |
| Verbal WM | 1 | 3 | -0.11 | -4.75 (95) | < .001 |
| Verbal WM | 1 | 4 | 0.02 | 0.47 (46) | 1.000 |
| Verbal WM | 1 | 5 | -0.01 | -0.27 (73) | 1.000 |
| Verbal WM | 2 | 3 | -0.06 | -3.00 (104) | .034 |
| Verbal WM | 2 | 4 | 0.07 | 1.75 (55) | .859 |
| Verbal WM | 2 | 5 | 0.04 | 1.90 (82) | .609 |
| Verbal WM | 3 | 4 | 0.13 | 2.89 (61) | .054 |
| Verbal WM | 3 | 5 | 0.10 | 4.36 (88) | < .001 |
| Verbal WM | 4 | 5 | -0.03 | -0.65 (39) | 1.000 |
| Reading ACC | 1 | 2 | 0.57 | 2.86 (89) | .052 |
| Reading ACC | 1 | 3 | 0.89 | 4.73 (95) | < .001 |
| Reading ACC | 1 | 4 | 0.46 | 1.05 (46) | 1.000 |
| Reading ACC | 1 | 5 | 0.56 | 2.52 (74) | .141 |
| Reading ACC | 2 | 3 | 0.32 | 2.08 (104) | .398 |
| Reading ACC | 2 | 4 | -0.11 | -0.31 (55) | 1.000 |
| Reading ACC | 2 | 5 | -0.01 | -0.07 (83) | 1.000 |
| Reading ACC | 3 | 4 | -0.43 | -1.31 (61) | 1.000 |
| Reading ACC | 3 | 5 | -0.34 | -1.97 (89) | .518 |
| Reading ACC | 4 | 5 | 0.09 | 0.26 (40) | 1.000 |
| Visual stm | 1 | 2 | -0.52 | -3.00 (89) | .035 |
| Visual stm | 1 | 3 | -0.72 | -3.85 (95) | .002 |
| Visual stm | 1 | 4 | 0.49 | 1.49 (46) | 1.000 |
| Visual stm | 1 | 5 | -0.15 | -0.72 (74) | 1.000 |
| Visual stm | 2 | 3 | -0.21 | -1.16 (104) | 1.000 |
| Visual stm | 2 | 4 | 1.01 | 3.06 (55) | .034 |
| Visual stm | 2 | 5 | 0.37 | 1.83 (83) | .711 |
| Visual stm | 3 | 4 | 1.21 | 3.15 (61) | .026 |
| Visual stm | 3 | 5 | 0.57 | 2.66 (89) | .091 |
| Visual stm | 4 | 5 | -0.64 | -1.57 (40) | 1.000 |
| Visual WM | 1 | 2 | -0.70 | -4.04 (89) | .001 |
| Visual WM | 1 | 3 | -0.85 | -4.40 (95) | < .001 |
| Visual WM | 1 | 4 | -0.07 | -0.23 (46) | 1.000 |
| Visual WM | 1 | 5 | -0.45 | -2.11 (74) | .384 |
| Visual WM | 2 | 3 | -0.15 | -0.80 (104) | 1.000 |
| Visual WM | 2 | 4 | 0.63 | 1.89 (55) | .644 |
| Visual WM | 2 | 5 | 0.25 | 1.18 (83) | 1.000 |
| Visual WM | 3 | 4 | 0.78 | 1.94 (61) | .575 |
| Visual WM | 3 | 5 | 0.40 | 1.74 (89) | .851 |
| Visual WM | 4 | 5 | -0.38 | -0.90 (40) | 1.000 |
| Numerical Fluency | 1 | 2 | -0.36 | -2.73 (87) | .077 |
| Numerical Fluency | 1 | 3 | -1.33 | -8.52 (93) | < .001 |
| Numerical Fluency | 1 | 4 | -0.05 | -0.22 (44) | 1.000 |
| Numerical Fluency | 1 | 5 | -0.76 | -4.67 (72) | < .001 |
| Numerical Fluency | 2 | 3 | -0.97 | -6.66 (104) | < .001 |
| Numerical Fluency | 2 | 4 | 0.30 | 1.15 (55) | 1.000 |
| Numerical Fluency | 2 | 5 | -0.40 | -2.58 (83) | .115 |
| Numerical Fluency | 3 | 4 | 1.27 | 3.80 (61) | .003 |
| Numerical Fluency | 3 | 5 | 0.57 | 3.18 (89) | .021 |
| Numerical Fluency | 4 | 5 | -0.70 | -2.12 (40) | .403 |
| Simple operation | 1 | 2 | -0.94 | -4.94 (88) | < .001 |
| Simple operation | 1 | 3 | -1.52 | -9.13 (95) | < .001 |
| Simple operation | 1 | 4 | -0.02 | -0.06 (46) | 1.000 |
| Simple operation | 1 | 5 | -0.84 | -3.96 (74) | .002 |
| Simple operation | 2 | 3 | -0.58 | -3.96 (103) | .001 |
| Simple operation | 2 | 4 | 0.92 | 2.68 (54) | .097 |
| Simple operation | 2 | 5 | 0.10 | 0.54 (82) | 1.000 |
| Simple operation | 3 | 4 | 1.49 | 5.37 (61) | < .001 |
| Simple operation | 3 | 5 | 0.67 | 4.23 (89) | < .001 |
| Simple operation | 4 | 5 | -0.82 | -2.26 (40) | .292 |
| Addition & Subtraction | 1 | 2 | -0.48 | -2.54 (84) | .129 |
| Addition & Subtraction | 1 | 3 | -1.32 | -8.07 (88) | < .001 |
| Addition & Subtraction | 1 | 4 | 0.21 | 0.63 (44) | 1.000 |
| Addition & Subtraction | 1 | 5 | -0.97 | -4.86 (69) | < .001 |
| Addition & Subtraction | 2 | 3 | -0.84 | -5.13 (96) | < .001 |
| Addition & Subtraction | 2 | 4 | 0.69 | 1.93 (52) | .586 |
| Addition & Subtraction | 2 | 5 | -0.49 | -2.46 (77) | .160 |
| Addition & Subtraction | 3 | 4 | 1.53 | 5.32 (56) | < .001 |
| Addition & Subtraction | 3 | 5 | 0.34 | 1.99 (81) | .496 |
| Addition & Subtraction | 4 | 5 | -1.18 | -3.39 (37) | .017 |
| Multiplication &  division | 1 | 2 | -0.38 | -2.25 (87) | .268 |
| Multiplication & | 1 | 3 | -1.26 | -7.71 (93) | < .001 |
| division | 1 | 4 | -0.41 | -1.38 (44) | 1.000 |
| Multiplication & | 1 | 5 | -0.84 | -4.65 (72) | < .001 |
| division | 2 | 3 | -0.87 | -5.30 (104) | < .001 |
| Multiplication & | 2 | 4 | -0.02 | -0.07 (55) | 1.000 |
| division | 2 | 5 | -0.45 | -2.38 (83) | .195 |
| Multiplication & | 3 | 4 | 0.85 | 2.55 (61) | .132 |
| division | 3 | 5 | 0.42 | 2.34 (89) | .216 |
| Multiplication & | 4 | 5 | -0.43 | -1.24 (40) | 1.000 |
| Estimation | 1 | 2 | -0.40 | -2.24 (88) | .276 |
| Estimation | 1 | 3 | -1.35 | -7.85 (93) | < .001 |
| Estimation | 1 | 4 | 0.22 | 0.69 (45) | 1.000 |
| Estimation | 1 | 5 | -0.82 | -4.37 (73) | < .001 |
| Estimation | 2 | 3 | -0.95 | -5.58 (103) | < .001 |
| Estimation | 2 | 4 | 0.62 | 1.77 (55) | .822 |
| Estimation | 2 | 5 | -0.42 | -2.21 (83) | .298 |
| Estimation | 3 | 4 | 1.56 | 4.65 (60) | < .001 |
| Estimation | 3 | 5 | 0.52 | 2.85 (88) | .055 |
| Estimation | 4 | 5 | -1.04 | -3.06 (40) | .040 |
| Numeration | 1 | 2 | -0.43 | -2.32 (88) | .226 |
| Numeration | 1 | 3 | -1.25 | -7.41 (94) | < .001 |
| Numeration | 1 | 4 | 0.16 | 0.44 (45) | 1.000 |
| Numeration | 1 | 5 | -0.71 | -3.36 (73) | .012 |
| Numeration | 2 | 3 | -0.82 | -5.02 (104) | < .001 |
| Numeration | 2 | 4 | 0.59 | 1.63 (55) | 1.000 |
| Numeration | 2 | 5 | -0.28 | -1.36 (83) | 1.000 |
| Numeration | 3 | 4 | 1.41 | 4.40 (61) | < .001 |
| Numeration | 3 | 5 | 0.54 | 2.91 (89) | .045 |
| Numeration | 4 | 5 | -0.87 | -2.17 (40) | .356 |
| Number line | 1 | 2 | 0.76 | 3.95 (89) | .002 |
| Number line | 1 | 3 | 0.79 | 4.75 (95) | < .001 |
| Number line | 1 | 4 | 0.16 | 0.37 (46) | 1.000 |
| Number line | 1 | 5 | 0.49 | 2.26 (74) | .269 |
| Number line | 2 | 3 | 0.03 | 0.23 (104) | 1.000 |
| Number line | 2 | 4 | -0.60 | -1.94 (55) | .579 |
| Number line | 2 | 5 | -0.27 | -1.61 (83) | 1.000 |
| Number line | 3 | 4 | -0.63 | -2.71 (61) | .088 |
| Number line | 3 | 5 | -0.30 | -2.19 (89) | .315 |
| Number line | 4 | 5 | 0.33 | 1.03 (40) | 1.000 |

Note. Group 1 = Reading Accuracy Difficulties (N = 41); Group 2 = Mild Mathematical Deficit (N = 48); Group 3 = High Mathematical Performance (N = 53); Group 4 = Inhibition Difficulties (N = 7); Group 5 = Average Mathematical Performance (N = 35). ΔM = mean difference. t values reported with df in parentheses. p values are Bonferroni-adjusted across all pairwise comparisons within each measure.

**Latent Class Analysis Profile Identification**

We conducted a Latent Class Analysis (LCA) to identify potential subgroups within our sample, using standardized indicators across domain-specific mathematics skills (e.g., math fluency, simple operations, addition/subtraction, multiplication/division, estimation, numeration, number line, symbolic/non-symbolic comparison RT/ACC) and domain-general abilities (e.g., verbal/visual working memory, vocabulary, reading accuracy/inhibition/coding, and Raven). BIC, entropy, and interpretability were used to compare 2–6 class solutions.

Four subgroups were identified through LCA. Table 1 shows the means and standard deviations for each subgroup's performance on all measures. Table 2 presents ANOVA results across LCA clusters, and Table 3 shows post-hoc comparisons for significant measures.

Four profiles were identified through Latent Class Analysis. Profile 1 (N=65) showed difficulties in inhibition, numeration, vocabulary, and mathematical operations. Profile 2 (N=59) demonstrated relatively strong performance across cognitive and mathematical domains, resembling the high-performing cluster (Group 3). Profile 3 (N = 32) exhibited strengths in mathematical areas, alongside some challenges in inhibition and visual memory. This profile did not correspond clearly to any cluster identified in the cluster analysis. Particular difficulties in continuous task accuracy characterized profile 4 (N=31).

Although these profiles provided initial insights, the resulting classifications showed considerable overlap in traits, making it difficult to distinguish clear subgroups or identify specific cognitive patterns. Therefore, we retained the cluster-analysis profiles as our primary solution.

**Table S2**

*Mean Z-scores for General and Specific Cognitive Domains across Clusters Identified by LCA*

|  | **Cluster 1** | **Cluster 2** | **Cluster 3** | **Cluster 4** |
| --- | --- | --- | --- | --- |
|  |  |  |  |  |
|  | N=79 | N=21 | N=32 | N=59 |
| **Math fluency test** | -0.69 (0.58) | -0.34 (0.63) | 0.63 (0.84) | -0.64 (0.77) |
| **Simple operations** | -0.54 (0.8) | 0.75 (0.45) | -0.15 (1) | -0.01 (1.1) |
| **Addition & Subtraction** | -0.73 (0.8) | 0.62 (0.7) | 0.56 (0.5) | -0.15 (0.9) |
| **Multiplication &**  **division** | -0.62 (0.76) | 0.42 (0.76) | 0.46 (0.83) | 0.08 (0.96) |
| **Estimation** | -0.88 (0.6) | 0.71 (0.6) | 0.73 (0.5) | 0.01 (0.9) |
| **Numeration** | -0.74 (0.76) | 0.62 (0.72) | 0.80 (0.54) | -0.20 (0.98) |
| **Non-symbolic comparison –RT** | 0.43 (1.06) | -0.13 (0.89) | -0.29 (0.98) | 0.76 (0.88) |
| **Symbolic comparison RT** | 0.13 (0.80) | -0.34 (0.54) | 0.44 (1.73) | -0.06 (0.93) |
| **Non-symbolic comparison –ACC** | 0.19 (0.8) | 0.66 (0.27) | -0.03 (0.8) | -1.79 (0) |
| **Number line** | 0.18 (0.98)- | 0.39 (0.48) | 0.20 (1.11)- | 0.15 (0.86)- |
| **Verbal fluency** | -0.29 (0.77) | 0.47 (0.93) | 0.04 (0.72) | -0.14 (0.88) |
| **Reading RT** | -0.14 (0.9) | -0.03 (0.9) | 0.3 (1.25) | 0.00 (1) |
| **Reading ACC** | 0.22 (1.02)- | 0.43 (0.58) | 0.18 (0.99)- | 0.07 (0.87)- |
| **Vocabulary** | -0.40 (0.8) | 0.63 (1) | -0.07 (0.92) | -0.24 (0.9) |
| **Verbal working memory** | 0.48 (0.10) | 0.58 (0.11) | 0.52 (0.09) | 0.52 (0.13) |
| **Short-term visual memory** | -0.27 (0.9) | 0.47 (0.7) | -0.30 (0.95) | 0.05 (1.1) |
| **Visual working memory** | -0.26 (0.9) | 0.44 (1) | 0.02 (1) | 0.00 (1) |
| **Inhibition** | -0.51 (1.74) | -0.18 (0.27) | 0.57 (1.4) | 0.07 (0.9) |
| **Coding** | -0.06 (0.27) | -0.17 (0.29) | -0.04 (0.28) | 0.14 (1.42) |
| **Non-verbal IQ** | -0.38 (0.80) | 0.30 (0.89) | -0.05 (1.09) | 0.07 (0.93) |

**Table S3**

| Variable | df | F-value | Eta Squared (η²) | p-value |
| --- | --- | --- | --- | --- |
| Continues ACC | F(3, 197) | 113.57 | 0.634 | < .001 |
| Number line | F(3, 197) | 5.96 | 0.083 | < .001 |
| Visual wm | F(3, 197) | 6.01 | 0.084 | < .001 |
| Math fluency test | F(3, 197) | 6.51 | 0.090 | < .001 |
| Raven | F(3, 186) | 6.69 | 0.097 | < .001 |
| Reading ACC | F(3, 197) | 6.93 | 0.095 | < .001 |
| Discreet RT | F(3, 197) | 8.68 | 0.117 | < .001 |
| Visual stm | F(3, 197) | 8.69 | 0.117 | < .001 |
| Verbal WM | F(3, 196) | 9.96 | 0.132 | < .001 |
| Vocabulary | F(3, 197) | 15.28 | 0.189 | < .001 |
| Multiplication &  division | F(3, 195) | 24.07 | 0.270 | < .001 |
| Simple operation | F(3, 196) | 26.42 | 0.288 | < .001 |
| Fluency Numerical | F(3, 195) | 32.33 | 0.332 | < .001 |
| Addition & Subtraction | F(3, 184) | 39.19 | 0.390 | < .001 |
| Numeration | F(3, 196) | 50.45 | 0.436 | < .001 |
| Estimation | F(3, 195) | 77.35 | 0.543 | < .001 |
| Fluency Non-Numerical | F(3, 197) | 10.00 | 0.132 | < .001 |
| Symbolic RT | F(3, 194) | 5.00 | 0.072 | = .002 |
| Flanker congruity RT | F(3, 196) | 4.20 | 0.060 | = .007 |
| Reading RT | F(3, 197) | 1.47 | 0.022 | = .224 |

*One-Way ANOVA: Between-Cluster Comparison*
