# Supplementary material for: Heterogeneity in mathematics: Investigating cognitive profiles and reading comorbidities among children
Source: Psychol Res. 2026 Feb 28;90(2):47. doi: 10.1007/s00426-026-02253-1 (PMC12950034; doi:10.1007/s00426-026-02253-1)
Supplement: Supplementary file 2 — Supplementary Material 2 (DOCX 32.4 KB) [file 426_2026_2253_MOESM2_ESM.docx]

Supplementary Materials for: Heterogeneity in Mathematics: Investigating Cognitive Profiles and Reading Comorbidities among Children.

Sonia Hasson, Sarit Ashkenazi

**Table S1**

Skewness and Kurtosis Indices for Study Measures

Table S1 presents skewness and kurtosis indices for all study measures, indicating the distribution characteristics of the data used in the analyses.

| **Measure** | **Skewness** | **Kurtosis** |
| --- | --- | --- |
| **Mathematical Measures** | | |
| KeyMath-3 (mean) | -0.04 | -0.61 |
| Arithmetic fluency | 0.63 | 0.30 |
| Addition accuracy | -1.49 | 1.80 |
| Multiplication accuracy | 0.11 | -1.12 |
| Subtraction accuracy | -1.15 | 0.48 |
| **Domain-General Measures** | | |
| Phonological fluency | 0.88 | 1.40 |
| Semantic fluency | -0.06 | 0.52 |
| Vocabulary | 0.68 | 0.71 |
| Coding speed | 0.92 | 1.17 |
| Raven matrices (non-verbal IQ) | -0.86 | 2.57 |
| Visual short-term memory | -0.23 | 0.05 |
| Verbal working memory | 0.22 | -0.17 |
| Visual working memory | 0.66 | 0.27 |
| **Reading** | | |
| Word reading speed | 1.05 | 1.41 |
| Word reading errors | 1.32 | 2.61 |
| Nonword reading speed | 1.11 | 2.62 |
| Nonword reading errors | 0.51 | -0.16 |

*Note*. Values represent skewness and kurtosis indices from the current sample (N = 186)
